# Educating Future Educators–Resident Distinction in Education: A Longitudinal Curriculum for Physician Educators

**DOI:** 10.5811/westjem.2021.11.53890

**Published:** 2021-12-17

**Authors:** Sandra Seelig, Erin Bright, Jessica Bod, David Della-Giustina, Katja Goldflam, Ryan F. Coughlin, Alina Tsyrulnik

**Affiliations:** Yale School of Medicine, Department of Emergency Medicine, New Haven, Connecticut

## BACKGROUND

The Accreditation Council for Graduate Medical Education (ACGME) lists “educating patients, families, students, residents, and other health professionals” as a common core requirement for residency programs in every medical specialty.^1^ Residents often play a crucial role in peer and medical student education. Teaching others can solidify resident knowledge, enhance students’ knowledge, and influence career choices.^2^,^3^,^4^,^5^

A 2004 review of existing resident-as-teacher (RAT) curricula outside of emergency medicine (EM) found that they improve resident teaching confidence, teaching organization, and student evaluations, as well as resident motivation to teach and confidence in their teaching skills.^6^ Unfortunately, many EM programs lack RAT curricula; a needs assessment revealed that 40% of EM programs lack RAT programming.^7^ Furthermore, many residents lack confidence in their teaching abilities.^8^ While bedside teaching curricula demonstrated improvement in the educational experience for both residents and medical students and an impact on residents’ pursuit of academic careers, EM RAT curricula are almost exclusively lecture-based.^9^,^10^,^11^ There is a paucity of literature describing longitudinal RAT programs that emphasize the large scope of skills EM residents need to be effective educators. Furthermore, programs that have been described have required financial and infrastructural support that may not be easily reproduced at other sites.^12^

## OBJECTIVES

Our objective was to create a longitudinal, multimodal RAT curriculum for EM residents that would foster early development of clinician-educators and develop residents’ confidence in their teaching abilities.

## CURRICULAR DESIGN

An elective curriculum, known as Resident Distinction in Education (RDE), was designed at our four-year residency using the six modules for creating an education curriculum described by Farrell et al: clinical teaching; bedside teaching; effective feedback; teaching procedures; teaching with high fidelity simulation; and leading effective lectures and discussions.^13^ A scholarly project requirement was included to address the importance of education-based scholarship for clinician-educators.^14^

To create the curriculum, we used pre-existing RAT opportunities within our residency program and added two that have been successful at other institutions. These were subdivided into three domains: teaching; scholarship; and personal learning/development. We assigned a numerical credit to activities proportional to the amount of time a resident was expected to spend in preparation and execution. Historically, prior to the creation of the RDE curriculum our program’s graduates would complete the equivalent of 74 credits over four years simply by participating in residency activities. This was used as a basis to set additional requirements for the RDE certification at 225 credits (Appendix A). We established annual credit requirements within each domain to help residents pace themselves. We then created an interactive spreadsheet listing requirements and credit designation in each domain to help residents track their progress and record the details of their experiences. Participants in the RDE program were all required to read the text *ABC of Learning and Teaching in Medicine*^15^ and to participate in journal clubs based on their reading. Two new RAT rotations were created: a clinical teaching elective focused on working with EM sub-interns, and a required bedside teaching rotation for senior residents. The RDE participants used these rotations to refine their skills by teaching procedures, participating in bedside teaching, and administering mini-lectures to residents working clinically in the emergency department (ED).

The RDE curriculum, introduced in 2018, is open for enrollment in the middle of the intern year as an elective. The directors meet with interested residents annually to track progress. Upon graduation, residents who fulfilled the RDE requirements received a certificate attesting to their dedication and recognizing their efforts and skills as an educator.

## IMPACT/EFFECTIVENESS

Since its implementation, 28 residents have enrolled in the RDE program. Eight of the enrolled residents have completed residency, and six satisfied all requirements to receive the RDE certificate. The two residents who did not complete the program failed to satisfy the requirements set forth. All six graduates who received the RDE certificate are currently working at academic institutions.

**Figure 1:**
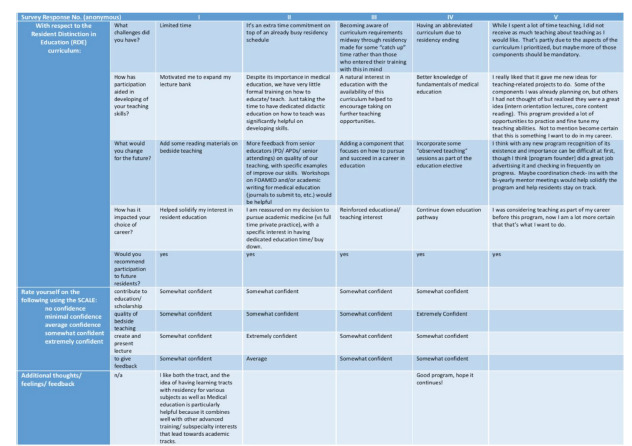
Survey results of graduates who completed the requirements for a Resident Distinction in Education certificate.* *The visual representation differs slightly from that of the survey collected for ease of reproduction in graphical form.

A survey that was deemed exempt by our institutional review board was sent to all graduates who completed the program; one graduate, a co-author of this study, was excluded (n = 5). We created the survey through our our university-based, web survey instrument (Qualtrics, Provo, UT). A link to the online survey was distributed via email to all residency graduates who had participated in the RDE program. No identifying information was collected from participants. The response rate was 100%. In evaluating the objective of fostering resident confidence in their teaching abilities, the survey results showed that all participants felt at least “somewhat confident” in their ability to contribute to education/ scholarship, quality of bedside teaching, and creating/ presenting lectures. All but one resident felt at least “somewhat confident” in giving feedback. “Extreme confidence” was reported by one resident in presenting lectures, and by another in bedside teaching. All respondents stated they would recommend participation in the program to future residents and that their involvement helped solidify their desire to actively incorporate medical education into their future careers (Figure 1).

The RDE program is currently in its third year of implementation, and RDE participants represent about one-third of each residency class. The RDE curriculum was designed to be reproducible within other EM residency programs. The ease of incorporating a similar program would depend on the breadth of pre-existing RAT opportunities at a given residency. However, some components likely exist at other programs, and minimal additional burden should be needed to implement a similar framework. Based on the limited number of participants, the one aspect of the RDE program that many have found particularly challenging and that led to two residents failing to complete the program was the scholarly project criteria. As of now, the definition of what constitutes a “scholarly project” at our institution is being evaluated and may be broadened.

We want to acknowledge several limitations. First, our program was implemented at a four-year residency with four months of elective time. This may limit reproducibility at programs with less elective time. One solution would be to decrease the total number of credits needed to attain the RDE at those programs. Another limitation is the lack of formal feedback or evaluation of participants’ bedside teaching and lecturing skills beyond self-assessment, which is prone to bias. The graduates rated themselves “average” or “somewhat competent” in their skills based on survey results. No self-evaluations were performed before participation in the program; thus, conclusions cannot be drawn as to the program’s effects on resident skills or level of comfort. The program affiliation with a medical school as well as a large university did provide our resident participants with many diverse teaching opportunities that could be difficult to replicate at programs that lack such affiliations.

The small number of participants did not allow for statistically significant conclusions to be drawn. The “soft” outcomes (Figure 1) based on self-reported confidence without true measures does demonstrate preliminary evidence of effectiveness based on the Kirkpatrick framework, level 1.^16^ The responses point to trainees finding their participation in the RDE program influential upon their development, all were “somewhat” or “extremely” confident in the domains evaluated by the survey (an achievement of Kirkpatrick level 1), and all pursued academic careers. Adding student and faculty evaluations of participant residents’ teaching skills, and pre-post self-assessments may allow us to gain a more thorough understanding of this program’s impact. Furthermore, tracking RDE graduates’ career trajectories will allow for conclusions regarding long-term impacts. These longer term and more thorough outcome measures would allow the evaluation of higher Kirkpatrick levels and enable a more robust evaluation of the RDE program’s success.

## Supplementary Information



## References

[1] Accreditation Council for Graduate Medical Education (2020). ACGME Common Program Requirements (residency).

[2] Geary A, Hess DT, Pernar LIM (2019). Resident-as-teacher programs in general surgery residency: a review of published curricula. Am J Surg.

[3] Schwartz MD, Linzer M, Babbott D (1991). Medical student interest in internal medicine. Initial report of the Society of General Internal Medicine Interest Group survey on factors influencing career choice in internal medicine. Ann Intern Med.

[4] Bing-You RG, Sproul MS (1992). Medical students’ perceptions of themselves and residents as teachers. Med Teach.

[5] Benè KL, Bergus G (2014). When learners become teachers: a review of peer teaching in medical student education. Fam Med.

[6] Wamsley MA, Julian KA, Wipf JE (2004). A literature review of “resident-as-teacher” curricula: Do teaching courses make a difference?. J Gen Intern Med.

[7] Ahn J, Jones D, Yarris LM (2017). A national needs assessment of emergency medicine resident-as-teacher curricula. Intern Emerg Med.

[8] Reyes C, Florenzano P, Contreras A (2012). A clinical teaching course for residents improves self-perception about preparation to teach. Rev Med Chil.

[9] Bod J, Tsyrulnik A, Coughlin R (2019). Successful Implementation of a resident liaison to medical students in emergency medicine rotations. AEM Educ Train.

[10] Jewett LS, Greenberg LW, Goldberg RM (1982). Teaching residents how to teach: a one-year study. J Med Educ.

[11] Ahn J, Golden A, Bryant A (2016). Impact of a Dedicated emergency medicine teaching resident rotation at a large urban academic center. West J Emerg Med.

[12] Heflin MT, Pinheiro S, Kaminetzky CP (2009). ‘So you want to be a clinician-educator...’: designing a clinician-educator curriculum for internal medicine residents. Med Teach.

[13] Farrell SE, Pacella C, Egan D (2006). Society for Academic Emergency Medicine, Undergraduate Education Committee. Resident-as-teacher: a suggested curriculum for emergency medicine. Acad Emerg Med.

[14] Ahn J, Martin SK, Farnan JM (2018). The graduate medical education scholars track: developing residents as clinician-educators during clinical training via a longitudinal, multimodal, and multidisciplinary track. Acad Med.

[15] Cantillon P, DF, Yardley S (2017). ABC of Learning and Teaching in Medicine.

[16] Kirkpatrick DL, Kirkpatrick JD (2006). Evaluating Training Programs: The Four Levels.

