# Supplementary figures and images for: Educating Future Educators–Resident Distinction in Education: A Longitudinal Curriculum for Physician Educators
Source: West J Emerg Med. 2021 Dec 17;23(1):100–2. doi: 10.5811/westjem.2021.11.53890 (PMC8782133; doi:10.5811/westjem.2021.11.53890)

Appendix A: RDE domains, teaching activities, and credit allotment


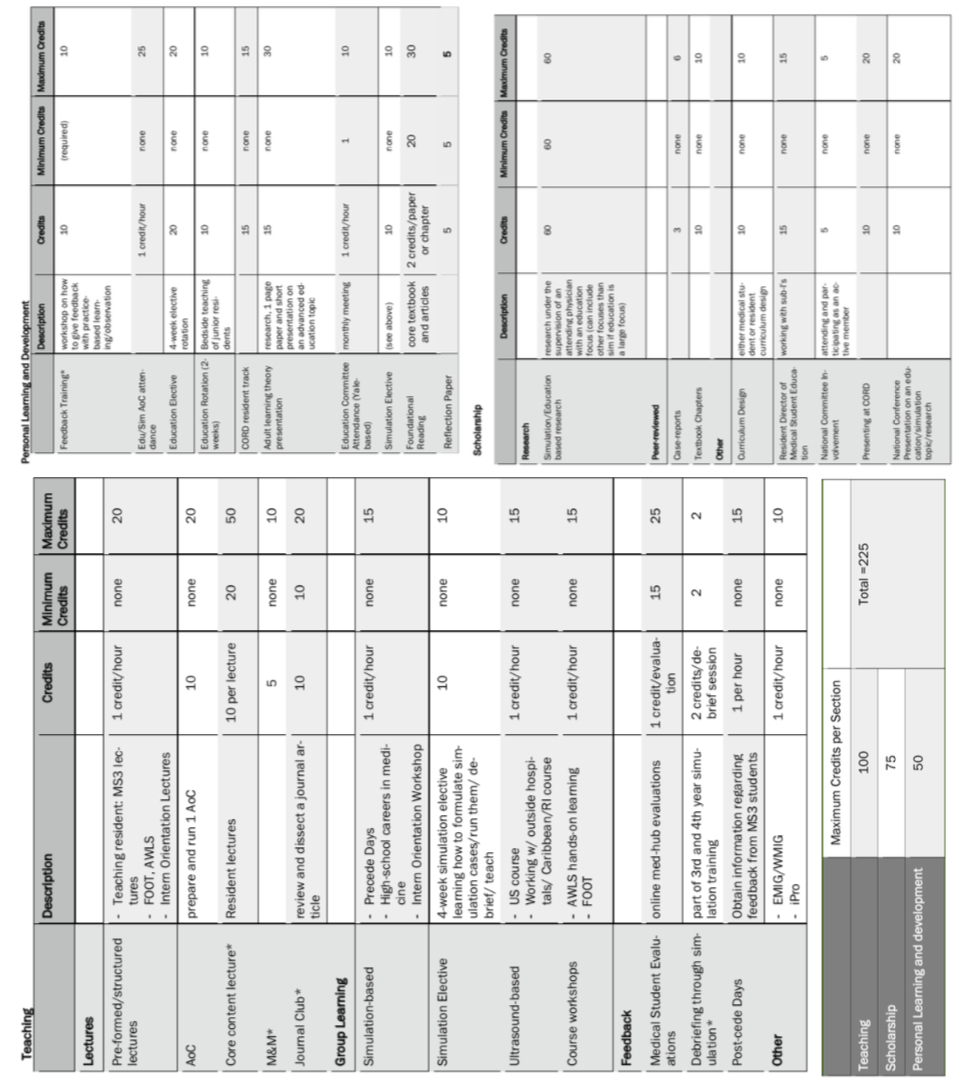

Supplement: Supplementary file 1 [file wjem-23-100-s001.docx]
